# Pre-pandemic family resources and child self-regulation in children’s internalizing problems during COVID-19: a multi-level social-ecological framework for emotional resilience

**DOI:** 10.3389/fpsyg.2023.1203524

**Published:** 2023-07-26

**Authors:** Luxi Chen, Wei-Jun Jean Yeung

**Affiliations:** ^1^Centre for Family and Population Research, National University of Singapore, Singapore, Singapore; ^2^Yong Loo Lin School of Medicine, National University of Singapore, Singapore, Singapore; ^3^Department of Sociology and Anthropology, National University of Singapore, Singapore, Singapore

**Keywords:** internalizing problems, economic stress, verbal cognitive ability, self-control, parental control, family resources, community resources, delay of gratification

## Abstract

**Introduction:**

Children’s psychological adjustment to adverse events can be determined by multiple risk and resilience factors. This study explored multi-level protective factors against children’s internalizing problems and investigated the mechanism regarding how diverse environmental and child-level resources influence children’s mental health in the context of COVID-19.

**Methods:**

Our participants included a nationally representative sample of 2,619 young children (48.3% girls) and their primary caregivers (95.1% mothers) in Singapore. They were a subset of the participants in the Singapore Longitudinal Early Development Study (SG LEADS). Data were collected over two waves—before the outbreak of the COVID-19 pandemic (Wave 1) when these children aged 3 to 6, and during the second year of the pandemic (Wave 2). Primary caregivers completed measures of verbal cognitive ability, self-control, economic stress, and positive and negative parental control in Wave 1. Children’s self-regulation was assessed by the Delay of Gratification task in Wave 1, and their internalizing problems were rated by their primary caregivers in both waves. Other pre-pandemic family and community characteristics were collected as covariates. Structural equation modeling was performed.

**Results:**

Pre-pandemic parental resources (i.e., verbal cognitive ability, self-control, and low economic stress) predicted children’s fewer internalizing problems during the pandemic and less aggravation of internalizing problems from before to during the pandemic, through more positive parental control (i.e., limit setting) and less negative parental control (i.e., harsh discipline). Moreover, children’s self-regulation during early childhood was predicted by their primary caregivers’ verbal cognitive ability and self-control, as well as positive parental control. Early childhood self-regulation further alleviated the aggravation of internalizing problems over time. Among the covariates, parental education, family income, parental psychological well-being, living with both parents, having a live-in domestic helper, and neighborhood quality also longitudinally predicted fewer child internalizing problems.

**Discussion:**

Our findings underscore the importance of nurturing children’s emotional resilience under adverse and uncertain circumstances by boosting protective factors in their social-ecological system, including community-, family-, parent-, and child-level resources.

## 1. Introduction

The global outbreak of the coronavirus disease (COVID-19) brought about unprecedented changes to individuals and families worldwide. Families with children have to adapt to the disruptions in their daily lives and routines in response to school closures, home-based learning, lack of social interactions, reduced outdoor activities, stay-at-home orders or quarantines, parents’ changes of work schedule, and their struggle between work and childcare. Children have been one of the most vulnerable populations to the negative impacts of COVID-19 (United Nations, 2020). They are generally more sensitive to these changes, which may impede their sense of predictability and security and subsequently impact their mental health (Wang et al., 2020). A large body of research has shown that children manifested an increase in externalizing symptoms (e.g., inattention, irritability, and hyperactivity) and internalizing symptoms (e.g., worry, fear, depression, and anxiety) during the pandemic across the globe (Crescentini et al., 2020; Cusinato et al., 2020; Duan et al., 2020; Francisco et al., 2020; Jiao et al., 2020; Bignardi et al., 2021; Di Giorgio et al., 2021; Khoury et al., 2021).

While environmental stressors may increase children’s risk of mental health problems, a wide array of protective factors can empower children to maintain or improve their well-being under uncertain and adverse circumstances. The process of using strengths, competencies, and resources to overcome contextual risks and maintain or enhance one’s well-being is broadly defined as resilience (Garmezy et al., 1984; Masten et al., 1990; Bonanno, 2004; Masten, 2014). The social-ecological framework of resilience emphasizes the crucial roles of individuals’ interactions with the environment (Ungar et al., 2013). Resilience can be promoted with protective factors related to individual differences, family contexts, and community characteristics (Bonanno, 2004). In other words, internal resources (such as personality traits, regulatory strategies, and developmental levels) and external resources (such as social support and interpersonal resources) should be utilized to foster children’s resilience. In the context of COVID-19, researchers have highlighted the importance of integrating multiple protective factors into studying resilience and longitudinal psychological outcomes (Chen and Bonanno, 2020). However, the complex mechanism regarding how diverse external and internal resources may work together to enhance children’s resilience and psychological adjustment to adverse events remains less well understood.

Therefore, it is essential to examine the pathways linking multi-level resources (e.g., community-, family-, parent-, and child-level resources) to children’s mental health during the COVID-19 pandemic. We aim to address this question in a socioeconomically and ethnically diverse sample of young children in Singapore—a high-income, highly educated, modern, multicultural, and multiracial country in South-East Asia. The nation experienced a prolonged period of COVID-19 lockdown with restrictions in 2020. The number of community cases grew dramatically in 2021. Survey data collected during pre-pandemic, pre-lockdown, and lockdown in Singapore has shown significant impacts of the COVID-19 pandemic on family income, childcare arrangements, family dynamics, and mental health (Yang et al., 2023). Nonetheless, a systematic investigation of children’s mental health and resilience in the context of COVID-19 in Singapore has been limited thus far compared to other countries.

According to a school-based survey on 2,139 children in Singapore (Woo et al., 2007), Singaporean children have higher rates of internalizing problems than externalizing problems, while Western children have higher rates of externalizing problems than internalizing problems in the non-COVID-19 context. While externalizing problems signify behaviors that are harmful and disruptive to others (such as aggression, oppositionality, and hyperactivity), internalizing problems are characterized by intropunitive emotions and moods, such as sadness, withdrawal, fear, and worry (Zahn-Waxler et al., 2000). A complex interplay between internal and environmental processes influences the emergence and changes in internalizing problems over time during childhood and adolescence (Zahn-Waxler et al., 2000). Hence, it is essential to explore environmental and individual-level protective factors against Singaporean children’s internalizing symptoms (e.g., anxiety, depression, and withdrawal) during the COVID-19 pandemic. This investigation can provide insight into how to promote Singaporean children’s emotional resilience under stressful and uncertain circumstances.

Family is the most proximal environment that influences early childhood development. According to the family stress theory, economic stress has negative impacts on children’s adjustment through disrupted parent–child interactions (Conger et al., 1994; Yeung et al., 2002; Masarik and Conger, 2017). Parents who experience higher economic stress tend to have lower psychological and relational resources. These parents may use less nurturing but more punitive parenting to discipline their children, which intensifies their children’s internalizing problems and externalizing problems (e.g., LaFrenière and Dumas, 1992; Linver et al., 2002; Yeung et al., 2002; Conger and Donnellan, 2007). Parents’ economic and psychological resources are crucial for children’s social-emotional development by enhancing functional parent–child interactions.

Furthermore, the lack of control in children’s early environment diminishes their sense of control and increases their psychological vulnerability to anxiety and depression (Chorpita and Barlow, 1998). It is noteworthy that different types of parental control can have dramatically different impacts on children’s social-emotional development. Harsh disciplinary strategies (such as criticism and aggressive or coercive behaviors) can be categorized as negative parental control, referred to as using a power-assertive method to excessively control children’s behaviors without granting age-appropriate autonomy. Negative parental control has been consistently associated with children’s poorer self-regulation (Blair and Raver, 2012) and more internalizing symptoms, particularly anxiety (Hudson and Rapee, 2002; Bayer et al., 2006). In contrast, positive parental control, with low to moderate power assertion (such as setting rules with guidance, instructions, and discussions with children), can have positive implications for children’s developmental outcomes, such as self-regulation (see Karreman et al., 2006, for a meta-analysis).

Self-regulation conceptualizes integrated processes to attain goals and manage significant life events and transitions (McClelland et al., 2010). Self-regulation has been a critical child-level protective factor in mitigating the impacts of contextual risks on children’s internalizing and externalizing problems (Lengua and Long, 2002; Lengua, 2003; Eiden et al., 2007; Lengua et al., 2008; Flouri et al., 2014). As an important aspect of self-regulation, Delay of Gratification (DoG) refers to the proclivity to forgo immediate and small gratification in order to attain more valuable but delayed rewards (Mischel, 1974; Mischel et al., 1989). Early DoG predicts children’s positive development in many domains, including more advanced social-emotional functioning, fewer behavior problems, greater cognitive functions, and school readiness across Western contexts (Mischel et al., 1989; Duckworth et al., 2013) and Asian contexts (Chen and Yeung, 2023a,b). Hence, nurturing children’s self-regulation during early childhood may improve their competence to adjust to stressful or challenging situations later in life.

During early childhood, self-regulation develops rapidly and adaptively based on early experiences (Eisenberg et al., 2005; Blair and Raver, 2012). According to the theory of self-regulation development, children progress from reactive, externally regulated, or co-regulated behavior to more advanced, proactive, or internally regulated behavior during the early years (Kopp, 1982; Diamond, 2002). The development of self-regulation, moving from external control (imposed by parents or caregivers) to internally controlling one’s emotional and behavioral impulses, is part of the socialization process in response to parent–child interactions (Calkins et al., 1998; Kochanska et al., 2000, 2001; Eisenberg et al., 2005). The meta-analysis conducted by Karreman et al. (2006) revealed that positive parental control (e.g., limit setting, guidance, and instructional behaviors) effectively fosters children’s self-regulation. In contrast, using harsh disciplinary strategies to overcontrol young children’s behaviors undermines children’s internalization of external controls and reduces their attempt to regulate their emotions and behaviors proactively, and consequently impedes their development of self-regulation (Silverman and Ragusa, 1992; Kochanska and Aksan, 1995; Calkins et al., 1998; Kochanska and Knaack, 2003).

Very few studies have incorporated the family stress theory and the model of self-regulation development in a single comprehensive framework to investigate children’s emotional and behavioral development. Prior research has demonstrated that contextual risk factors (such as economic disadvantages or adverse life events) lead to dysfunctional parenting behaviors (e.g., punitive or inconsistent discipline, lower responsiveness, and less support for autonomy), which further result in children’s poorer self-regulation (Lengua, 2009; Hardaway et al., 2012). Furthermore, children’s compromised self-regulation mediates the impact of economic disadvantages on their social-emotional functioning (see Raver, 2004 for a review). In particular, the mediating role of self-regulation in the longitudinal relations of family functioning and parenting behaviors to children’s externalizing problems has been well-documented in the literature (Eisenberg et al., 2005; Valiente et al., 2006; Hardaway et al., 2012). Nevertheless, there has been relatively less direct evidence for the mediating role of self-regulation in the longitudinal associations between family processes (including parenting) and children’s internalizing problems. The examination of the mediating pathways linking family resources, positive and negative parental control, and child self-regulation to children’s internalizing problems is thus needed.

Another limitation in the literature is that very few studies have systematically investigated the influences of parental resources in various forms (e.g., cognitive, psychological, and economic resources) on parenting strategies and children’s adjustment. Family functioning and parenting usually derive from parental characteristics, personality, resources, and competence (Chen and Luster, 2002). Parental self-control (the ability to regulate one’s cognition, emotions, and voluntary behaviors in accordance with internal goals) and verbal cognitive ability may play crucial roles in nurturing children’s self-regulation and emotional development. Parents higher on self-control in their daily lives (e.g., breaking bad habits, resisting temptations, and regulating emotions) may tend to set clearer rules for their children’s activities. These parents may also be able to inhibit the tendency to use emotionally charged or harsh disciplinary strategies to discipline children, which are harmful to children’s internalization of the rules. With more advanced verbal cognitive ability, parents can use better reasoning and richer vocabulary to guide, teach, and encourage their children to regulate their behaviors to meet changing situational demands. Teaching-based control, expressivity, guidance, and appropriate instructions can facilitate children to regulate their behaviors and emotions proactively (Olson et al., 1990; Calkins et al., 1998; Kochanska et al., 2000; Putnam et al., 2002; Eiden et al., 2007; Lengua et al., 2007). It is reasonable to expect that verbal cognitive ability and self-control can provide parents with cognitive and psychological resources to engage in functional parental control, nurture children’s self-regulation, and enhance their emotional resilience.

Taken together, the overarching aim of the present study was twofold: (1) to identify multi-level protective factors against young children’s internalizing problems and (2) to investigate the mechanism regarding how primary caregivers’ cognitive, psychological, and economic resources may influence young children’s internalizing problems through parental control and child self-regulation. Other family and community characteristics, such as parental education, family income, parental psychological well-being, living arrangements, and neighborhood quality, were included as covariates in this study. To increase the generalizability of the findings, the current investigation uses a large and nationally representative sample of young children in Singapore as an example. By addressing these aims in the context of COVID-19, the findings can advance the understanding of Asian children’s emotional resilience from the social-ecological perspective.

**Figure 1:**
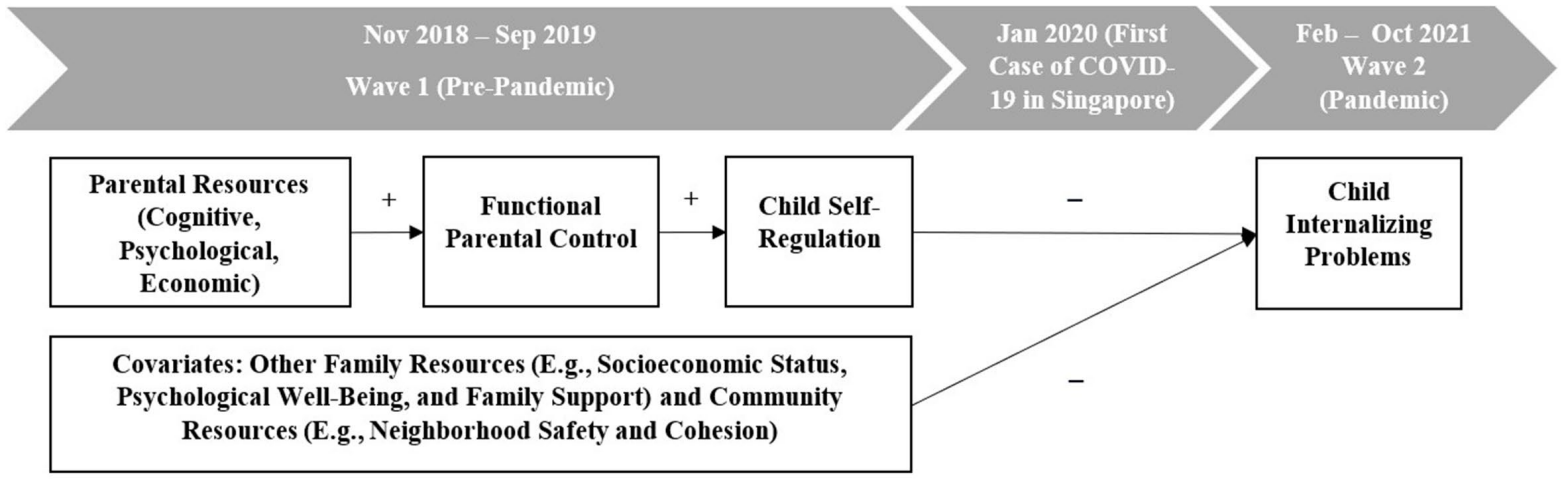
Proposed pathways linking multi-level resources to child internalizing problems.

Figure 1 displays the proposed mediating pathways linking multi-level resources to child internalizing problems. We posit that primary caregivers’ cognitive, psychological, and economic resources would predict children’s fewer internalizing problems during the COVID-19 pandemic by enhancing functional parental control and improving children’s self-regulation. Specifically, we proposed the following hypotheses:

*Hypothesis 1a*: Parental resources (e.g., verbal cognitive ability, self-control, and less economic stress) would predict fewer child internalizing problems.

*Hypothesis 1b*: Functional parental control (e.g., more positive parental control and less negative parental control) would predict fewer child internalizing problems.

*Hypothesis 1c*: Child self-regulation (e.g., DoG) would predict fewer child internalizing problems.

*Hypothesis 1d*: Other family and community resources (as covariates in this study), such as parental education, family income, parental psychological well-being, living with two parents, having a live-in helper, living with grandparents, and neighborhood quality, would predict fewer child internalizing problems.

*Hypothesis 2a*: Parental control would mediate the longitudinal relations of parental resources to children’s internalizing problems.

*Hypothesis 2b*: Child self-regulation would mediate the longitudinal relations of parental resources and parental control to children’s internalizing problems.

*Hypothesis 3a*: Parental control would mediate the effect of parental resources on changes in child internalizing problems over time.

*Hypothesis 3ba*: Child self-regulation would mediate the effects of parental resources and parental control on changes in child internalizing problems over time.

## 2. Methods

### 2.1. Participants and procedure

Our participants included a nationally representative sample of 2,619 young children in Singapore (48.3% girls) and their primary caregivers (95.1% were mothers, 3.7% were fathers, and 1.2% were others). They were a subset of the participants from the Singapore Longitudinal Early Development Study (SG LEADS; Yeung et al., 2020, 2022), carried out by the authors’ research team. A total of 5,005 children under age 7 took part in the first wave of SG LEADS. Only children aged 3 years and above (*n* = 2,973 in Wave 1) were eligible for the measures of self-regulation and internalizing problems. Finally, 2,619 children attended child assessments in both waves and became the final sample in our current research. Child-level normalized sampling weight was applied to all analyses to account for the initial selection probability. Among these children, 66.8% were ethnic Chinese, 15.8% were Malays, 12.3% were Indians, and 5.1% were from other ethnic backgrounds.

Data were collected over two waves during home visits. The first wave of data collection was conducted from November 2018 to September 2019, about 4 to 14 months before the outbreak of the COVID-19 pandemic. Children aged between 36 and 83 months (*M_wave1age_* = 58.9, *SD_wave1age_* = 14.1) in Wave 1. Children completed the DoG task, which measured self-regulation. Primary caregivers responded to measures on family and community characteristics, verbal cognitive ability, self-control, economic stress, parenting strategies, and child internalizing problems. The second wave of data collection was conducted during the second year of the pandemic, from February to October 2021. The average interval between the two waves was 24.9 months (*SD* = 3.21, *range* = 13–38). These children became 55 to 118 months old (*M_wave2age_* = 83.8, *SD_wave2age_* = 14.3). Primary caregivers rated child internalizing problems again in Wave 2. This study was approved by the Institutional Review Board (IRB) at the National University of Singapore (Approval code: S-17-326).

### 2.2. Measures

#### 2.2.1. Parental verbal cognitive ability

We selected eight items from the Passage Comprehension Test in the Woodcock-Johnson IV Tests of Achievement (WJ IV ACH; McGrew et al., 2014; Schrank et al., 2014) to measure primary caregivers’ verbal cognitive ability. Correct response scored 1, and no response or error scored 0. Scores of all items were summed to indicate verbal cognitive ability (Cronbach’s alpha was 0.83 in the current sample), with a higher score indicating more advanced verbal cognitive ability.

#### 2.2.2. Parental self-control

Parental self-control was measured by 10 items selected and modified from the Brief Self-Control Scale (BSCS; Tangney et al., 2004). Three items were positively keyed (e.g., “I refuse things that are bad for me”), and seven items were negatively keyed (e.g., “Sometimes I cannot stop myself from doing something, even if I know it is wrong” and “Pleasure and fun sometimes keep me from getting work done”). Primary caregivers reported the extent to which each statement described them on a 5-point scale ranging from 1 (not at all like me) to 5 (very much like me). We reversed the scoring of all negatively keyed items and then averaged the scores of all 10 items to indicate self-control (Cronbach’s alpha was 0.77 in the current sample). A higher score indicates greater self-control.

#### 2.2.3. Economic stress

One single item, “At the end of the month, do you (and your family) usually end up with some money left over, just enough to make ends meet, or not enough money to make ends meet?” was used to measure family economic stress (1 = “some money leftover,” 2 = “just enough to make ends meet / just enough to cover all expenses,” and 3 = “not enough to make ends meet /not enough to cover expenses”). A higher score indicates a higher level of economic stress.

#### 2.2.4. Positive parental control (limit setting)

Primary caregivers reported how often they set limits on their children’s activities in the past month, including setting limits on “how late your child(ren) can stay up at night,” “how much candy, sweets, or other snacks your child(ren) can have,” “which other children your child(ren) spend(s) time with,” “set a time when your child(ren) do(es) homework,” and “how your child(ren) spend(s) time after school or daycare,” as well as “discuss these rules with your child(ren).” The 5-point scale ranges from 1 (never) to 5 (very often). Scores of all six items were averaged to indicate positive parental control (Cronbach’s *α* = 0.79), with a higher score indicating more positive parental control.

#### 2.2.5. Negative parental control (harsh discipline)

Primary caregivers reported how often they disciplined their children in the past month, using high power-assertiveness methods, such as physical punishment (e.g., “spanking” and “grounding”), scolding (e.g., “had to scold or threaten your child for misbehavior”), taking away privileges (e.g., “taking away privileges”), and time-out (e.g., “sending the child to his/her room”). The 5-point scale ranges from 1 (not in the past month) to 5 (every day). The average score of all six items was computed to indicate negative parental control (Cronbach’s *α* = 0.60), with a higher score indicating more negative parental control.

#### 2.2.6. Child DoG

We modified Prencipe and Zelazo’s (2005) standard DoG choice task to measure children’s DoG. Nine test trials were created by crossing three types of reward (i.e., balloons, stickers, and erasers) and three types of choice (i.e., 1 now vs. 2 later, 1 now vs. 4 later, and 1 now vs. 6 later). Each child was presented with the actual rewards for both “now” or “later” options (e.g., 1 balloon for the “now” option and 4 balloons for the “later” option) and asked to choose between the two options. During each test trial, the “now” option scored 0, and the child would receive the small reward immediately. The “later” option scored 1, and the larger reward would be put into an envelope, set aside, and received by the child after the game, which took about 10 min. Scores of all nine test trials were summed to indicate the child’s DoG, with a higher score indicating a greater ability to delay gratification. The choice paradigm has shown good reliability, convergent validity, and predictive validity in a large and nationally representative sample of Singaporean young children (Chen & Yeung, 2023a).

#### 2.2.7. Child internalizing problems

Children’s internalizing problems (such as withdrawal, anxiety, and depression) were measured by 13 items selected from the Behavior Problems Index (BPI) developed by Peterson and Zill (1986) and based on earlier work by Achenbach and Edelbrock (1981). The items used in this study were identified by the factor analysis conducted in the current sample (see Appendix A in the Supplementary materials). The internalizing problems subscale possessed good internal reliability in the current sample (Cronbach’s alphas were 0.83 and 0.85 in Wave 1 and Wave 2, respectively). The primary caregiver reported the child’s behavior on a 3-point scale (1 = “often true,” 2 = “sometimes,” and 3 = “not true”). We recoded the responses as 0 = “not true,” 1 = “sometimes,” and 2 = “often true.” Scores of all relevant items were summed to indicate internalizing problems, with a higher score indicating more internalizing problems.

#### 2.2.8. Controls

##### 2.2.8.1. Child demographics

Child gender (dummy coded as 1 = girl, 0 = boy), ethnicity (three dummy variables were created, namely Malays, Indians, and Others, with Chinese as the reference group), and child age (in months) in both waves were collected.

##### 2.2.8.2. Parental education

Primary caregivers reported their educational attainment. Parental education is classified into three categories, namely Low Education (no formal schooling, primary school, or secondary school), Medium Education (post-secondary non-tertiary general or vocational education, polytechnic diploma, professional qualification, or other diploma), and High Education (Bachelor’s, postgraduate diploma, or Master’s and Doctorate or equivalent).

##### 2.2.8.3. Annual household income per capita

Primary caregivers reported their household income in the past 12 months. Annual household income per capita was calculated by dividing the total annual household income by the number of family members residing in the household. Annual household income per capita was log-transformed for analysis in this study.

##### 2.2.8.4. Parental psychological distress

The 6-item Kessler Psychological Distress Scale (K6) was deployed by Kessler et al. (2002) to assess non-specific psychological distress. Primary caregivers reported the frequency of feeling “nervous,” “hopeless,” “restless or fidgety,” “that everything was an effort,” “so sad that nothing could cheer you up,” and “worthless” in the past 4 weeks, on a 5-point scale where 1 indicates “all of the time” and 5 indicates “none of the time.” We recoded the response as 0 = “none of the time,” 1 = “a little of the time,” 2 = “some of the time,” 3 = “most of the time,” and 4 = “all of the time.” Scores of all items were summed to indicate the level of non-specific psychological distress (Cronbach’s *α* = 0.86 in the current sample), with a higher score indicating a higher level of psychological distress.

##### 2.2.8.5. Single parenthood

Based on the primary caregiver’s marital status, a dummy variable was created to indicate single parenthood (1 = single-parent, 0 = two-parent). Responses of “never married,” “divorced,” and “widowed” were recoded as “1,” and “currently married” was recoded as “0.”

##### 2.2.8.6. Living arrangements

We collected information about all the members living in the household. Two dummy variables were created, namely having a live-in domestic helper (1 = at least one live-in domestic helper, 0 = no live-in domestic helpers) and living with grandparents (1 = living with at least one grandparent, 0 = living without grandparents).

##### 2.2.8.7. Neighborhood quality

Primary caregivers rated the quality of the neighborhood (considered 15 to 20 min walking distance from the house) on six items. The first item was a general rating of the neighborhood as a place to raise children on a 5-point scale ranging from 1 = “poor” to 5 = “excellent.” The second item concerned the safety of walking around alone in the neighborhood after dark on a 4-point scale ranging from 1 = “extremely dangerous” to 4 = “completely safe.” The third to sixth items measured the characteristics of the neighbors, including friendliness, taking care of each other, trust on each other, and familiarity with each other, on a 7-point scale, with a higher score indicating a higher level of each characteristic. The z-score was computed for each item. The z-scores of all six items were averaged to indicate the perceived quality of the neighborhood (Cronbach’s *α* = 0.82), with a higher score indicating better quality of the neighborhood.

### 2.3. Analytics strategy

Descriptive statistics and bivariate correlations among all variables were calculated. A series of Structural Equation Models (SEMs) was performed on Mplus 7.31 (Muthén and Muthén, 1998) to (1) examine the longitudinal relations of diverse pre-pandemic environmental and child-level resources to children’s internalizing problems during the COVID-19 pandemic, (2) establish the mediating pathways linking pre-pandemic parental resources, parental control, and child self-regulation to child internalizing problems during the pandemic, when controlling for other pre-pandemic family and community characteristics, and (3) test the pathways to changes in child internalizing problems over time, when controlling for pre-pandemic child internalizing problems. Root Mean Square Error of Approximation (RMSEA), Comparative Fit Index (CFI), Tucker–Lewis Index (TLI), and Standardized Root Mean Square Residual (SRMR) were presented to indicate model fit. Normalized child-level sampling weight was applied to all analyses.

## 3. Results

### 3.1. Preliminary analyses: descriptive statistics, bivariate correlations, and changes in children’s internalizing problems from wave 1 to wave 2

Descriptive statistics of all main variables and bivariate correlations between Wave 1 variables and Wave 2 child internalizing problems are presented in Table 1. The paired sample *T*-test was performed to compare child internalizing problems measured in two waves. Based on primary caregivers’ ratings, children displayed more internalizing problems during the COVID-19 pandemic than before the outbreak of the pandemic, *t*(2813) = 13.7, *p* < 0.001.

**Table 1:** Descriptive statistics of all main variables and bivariate correlations of wave 1 (W1) variables with wave 2 (W2) child internalizing problems.

	*r*	*M*	*SD*	*Range*	*N*
W2 Child Internalizing Problems	–	2.97	3.33	0–24	2,619
W1 Parental Verbal Cognitive Ability	−0.15^***^	3.97	2.39	0–8	2,619
W1 Parental Self-Control	−0.063^**^	4.00	0.55	1.6–5	2,619
W1 Economic Stress	0.11^***^	1.43	0.62	1–3	2,619
W1 Positive Parental Control	−0.071^***^	3.30	0.74	1–5	2,458
W1 Negative Parental Control	0.065^***^	1.72	0.54	1–4.2	2,619
W1 Child Delay of Gratification	−0.019	5.19	3.62	0–9	2,604
Controls					
W1 Child Internalizing Problems	0.24^***^	1.94	3.19	0–26	2,601
W1 Child Age (in months)	0.080^***^	58.9	14.1	36–83	2,619
W2 Child Age (in months)	0.097^***^	83.8	14.3	55–118	2,619
Child Gender					
Girl (%)	0.002	48.3	–	–	2,619
Boy (%)	−0.002	51.7	–	–	2,619
Child Ethnicity					
Chinese (%)	0.041^*^	66.8	–	–	2,619
Malays (%)	0.039^*^	15.8	–	–	2,619
Indians (%)	−0.049^**^	12.3	–	–	2,619
Others (%)	−0.078^***^	5.1	–	–	2,619
Parental Education					
High (%)	−0.13^***^	48.1	–	–	2,619
Medium (%)	0.059^**^	29.5	–	–	2,619
Low (%)	0.088^***^	22.4	–	–	2,619
W1 Annual Household Income Per Capita (Log)	−0.11^***^	4.17	0.62	0–6.02	2,582
W1 Parental Psychological Distress	0.11^***^	3.03	3.67	0–22	2,619
W1 Single Parenthood (%)	0.062^**^	3.97	−	−	2,619
W1 Living with Grandparent(s) (%)	0.078^***^	23.1	−	−	2,619
W1 Having a Live-In Domestic Helper(s) (%)	−0.068^***^	34.0	−	−	2,619
W1 Neighborhood Quality (Z-Score)	−0.054^**^	0.000	0.72	−3.85–1.91	2,619

*** *p* < 0.001;

** *p* < 0.01;

* *p* < 0.01.

### 3.2. SEM: pathways linking pre-pandemic parental resources, parental control, and child self-regulation to child internalizing problems during the pandemic

In the first model, we examined the direct effect of pre-pandemic parental resources on children’s internalizing problems during the COVID-19 pandemic. Primary caregivers’ verbal cognitive ability and self-control before the pandemic predicted fewer child internalizing problems during the pandemic (*β* = −0.057, *SE_β_* = 0.031, *p* = 0.062, 95% CI[−0.11, −0.007], and *β* = −0.13, *SE_β_* = 0.043, *p* = 0.002, 95% CI[−0.21, −0.062], respectively). However, pre-pandemic economic stress did not directly affect children’s internalizing problems during the pandemic (*β* = 0.065, *SE_β_* = 0.058, *p* = 0.26, 95% CI[−0.030, 0.16]).

A set of covariates (i.e., child age, gender, ethnicity, parental education, parental psychological distress, annual household income per capita, single parenthood, having a live-in domestic helper, living with grandparents, and neighborhood quality) were entered into the second model. The model exhibited good model fit (RMSEA = 0.039, 90% CI [0.031, 0.048], CFI = 0.99, TLI = 0.98, SRMR = 0.024). The direct effect of primary caregivers’ verbal cognitive ability on child internalizing problems remained significant (*β* = −0.11, *SE_β_* = 0.021, *p* < 0.001, 95% CI[−0.14, −0.072]), but the direct effect of primary caregivers’ self-control on child internalizing problems became nonsignificant (*β* = −0.015, *SE_β_* = 0.016, *p* = 0.34, 95% CI[−0.042, 0.011]). The direct effect of pre-pandemic economic stress on child internalizing problems during the pandemic remained nonsignificant (*β* = 0.012, *SE_β_* = 0.016, *p* = 0.45, 95% CI[−0.014, 0.038]).

**Figure 2:**
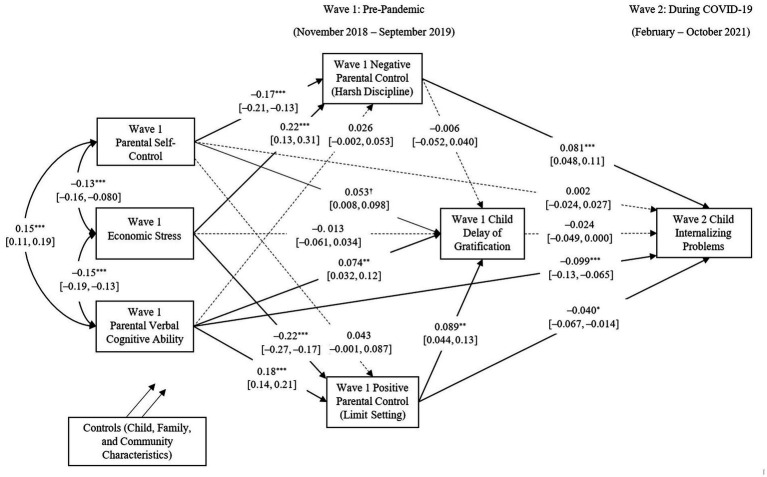
Pre-pandemic parental resources predict children’s internalizing problems during the COVID-19 pandemic through parental control. Note: Covariates included child age, gender, ethnicity, parental education, annual household income per capita, parental psychological distress, single parenthood, living with grandparent(s), having a live-in domestic helper(s), and neighborhood quality. Bold lines indicate significant paths, normal lines indicate marginally significant paths, and dotted lines indicate nonsignificant paths. 95% confidence intervals are presented in square brackets. ***p < 0.001, **p < 0.01, *p < 0.05, and ^†^*p* < 0.10.

In the third model, positive and negative parental control and child DoG were entered as the mediators. The model obtained adequate model fit (RMSEA = 0.050, 90% CI [0.043, 0.058], CFI = 0.97, TLI = 0.84, SRMR = 0.022). As illustrated in Figure 2, primary caregivers’ verbal cognitive ability directly predicted fewer child internalizing problems, and this relationship was also mediated by positive parental control (indirect effect: *β* = −0.007, *SE_β_* = 0.003, *p* = 0.019). Primary caregivers’ self-control indirectly predicted fewer child internalizing problems through less negative parental control (indirect effect: *β* = −0.014, *SE_β_* = 0.004, *p* = 0.001). Economic stress had an indirect effect on child internalizing problems through more negative parental control (indirect effect: *β* = 0.018, *SE_β_* = 0.006, *p* = 0.002) and less positive parental control (indirect effect: *β* = 0.009, *SE_β_* = 0.004, *p* = 0.018). Among the covariates, parental psychological distress, single parenthood, and living with grandparents predicted more child internalizing problems during the pandemic. In contrast, parental education, annual household income per capita, having a live-in domestic helper, and neighborhood quality predicted fewer child internalizing problems during the pandemic. All factors accounted for 69.7% of the variation in children’s internalizing problems during COVID-19.

**Figure 3:**
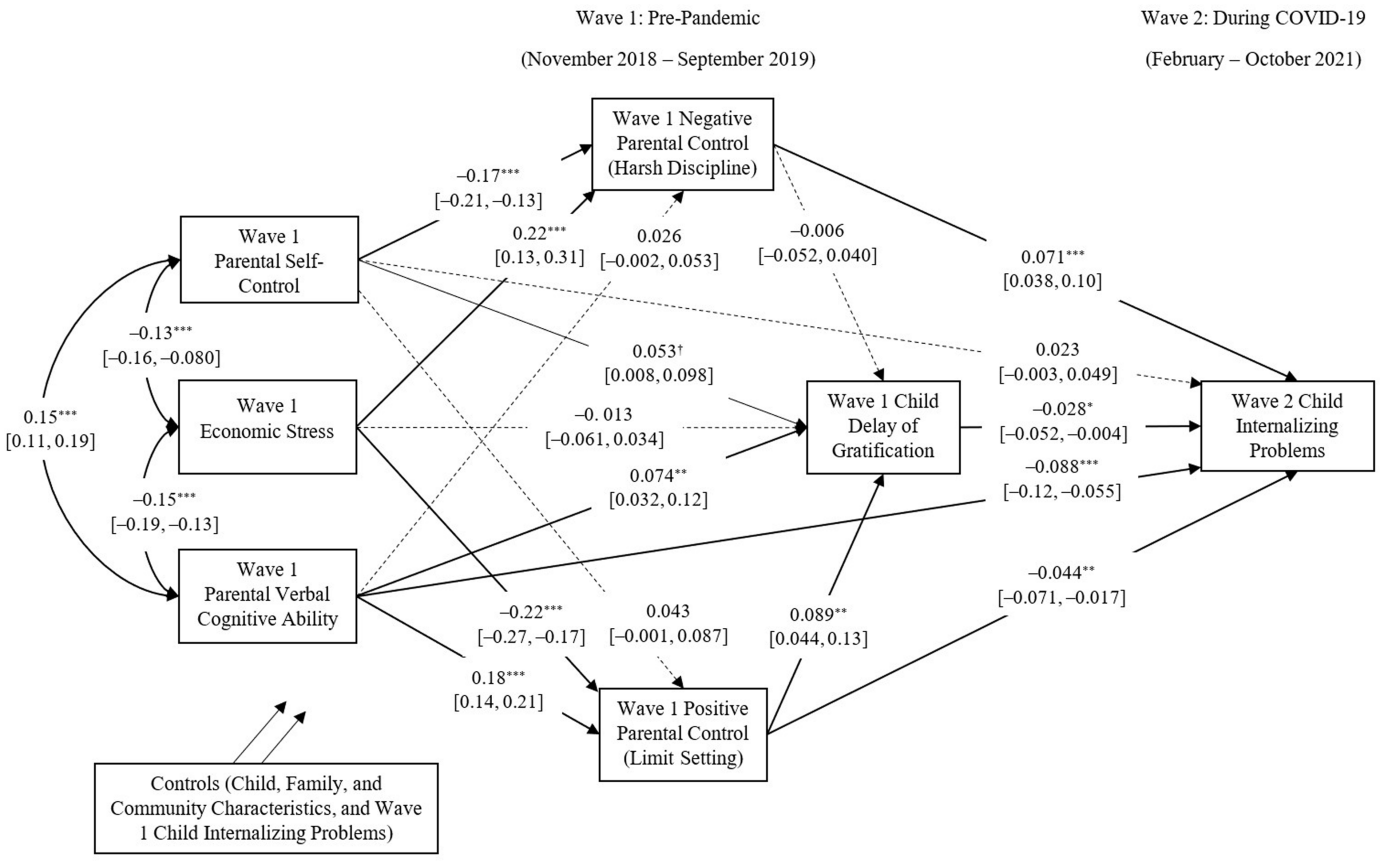
Pre-pandemic parental resources predict changes in child internalizing problems from Wave 1 (pre-pandemic) to Wave 2 (during the COVID-19 pandemic) through parental control and child self-regulation. Note: Covariates included pre-pandemic internalizing problems, child age, gender, ethnicity, parental education, annual household income per capita, parental psychological distress, single parenthood, living with grandparent(s), having a live-in domestic helper(s), and neighborhood quality. Bold lines indicate significant paths, normal lines indicate marginally significant paths, and dotted lines indicate nonsignificant paths. 95% confidence intervals are presented in square brackets. ***p < 0.001, **p < 0.01, *p < 0.05, and ^†^*p* < 0.10.

The final model controlled for pre-pandemic child internalizing problems to examine the pathways to changes in child internalizing problems from before to during the pandemic. The data fit the model well (RMSEA = 0.055, 90% CI[0.049, 0.061], CFI = 0.95, TLI = 0.78, SRMR = 0.038). As shown in Figure 3, primary caregivers’ verbal cognitive ability directly and negatively predicted children’s increases in internalizing problems from before to during the pandemic, and this relation was also mediated by positive parental control (indirect effect: *β* = −0.008, *SE_β_* = 0.003, *p* = 0.010). Primary caregivers’ self-control indirectly and negatively predicted children’s aggravation of internalizing problems over time through less negative parental control (indirect effect: *β* = −0.012, *SE_β_* = 0.004, *p* = 0.003). Economic stress had an indirect effect on children’s increases in internalizing problems over time through more negative parental control (indirect effect: *β* = 0.015, *SE_β_* = 0.005, *p* = 0.003) and less positive parental control (indirect effect: *β* = 0.010, *SE_β_* = 0.004, *p* = 0.010). Moreover, children’s DoG during early childhood was predicted by concurrently measured primary caregivers’ self-control, verbal cognitive ability, and positive parental control. Early childhood DoG further reduced children’s aggravation of internalizing problems over time. Among the covariates, single parenthood and living with grandparents predicted a larger increase in child internalizing problems from before to during the pandemic. In contrast, parental education, annual household income per capita, and having a live-in domestic helper predicted less aggravation of child internalizing problems over time. All variables explained 70.7% of the variation in Wave 2 internalizing problems (*ps* < 0.001).

Table 2 details the standardized coefficients of the effects of all main variables in Wave 1 on child internalizing problems in Wave 2 and changes in child internalizing problems from Wave 1 to Wave 2. The indirect effects of parental resources through parental control and child self-regulation are presented in Table 3.

**Table 2:** Effects of pre-pandemic variables in wave 1 (W1) on child internalizing problems during COVID-19 in wave 2 (W2) and changes in child internalizing problems from W1 to W2.

	Model 3 (Child Internalizing Problems in W2)			Model 4 (Changes in Child Internalizing Problems from W1 to W2)		
	*β*	*SE_β_*	*95% CI*	*β*	*SE_β_*	*95% CI*
Predictors						
W1 Parental Verbal Cognitive Ability	−0.099^***^	0.021	[−0.13, −0.065]	−0.088^***^	0.020	[−0.12, −0.055]
W1 Parental Self-Control	0.002	0.016	[−0.024, 0.027]	0.023	0.016	[−0.003, 0.049]
W1 Economic Stress	−0.015	0.016	[−0.041, 0.011]	−0.019	0.016	[−0.045, 0.006]
Mediators						
W1 Positive Parental Control	−0.040^*^	0.016	[−0.067, −0.014]	−0.044^**^	0.016	[−0.071, −0.017]
W1 Negative Parental Control	0.081^***^	0.020	[0.048, 0.11]	0.071^***^	0.020	[0.038, 0.10]
W1 Child Delay of Gratification	−0.024	0.015	[−0.049, <0.001]	−0.028^*^	0.015	[−0.052, −0.004]
Controls						
W1 Child Internalizing Problems	−	−	−	0.13^***^	0.033	[0.072, 0.18]
W1 Child Age (in months)	−0.007	0.067	[−0.12, 0.11]	0.037	0.067	[−0.073, 0.15]
W2 Child Age (in months)	0.072	0.076	[−0.052, 0.20]	0.019	0.073	[−0.10, 0.14]
Child Gender: Girl (Ref: Boy)	0.007	0.013	[−0.015, 0.028]	0.008	0.013	[−0.013, 0.030]
Child Ethnicity (Ref: Chinese)						
Malays	0.020	0.014	[−0.002, 0.042]	0.019	0.013	[−0.003, 0.040]
Indians	−0.033^*^	0.015	[−0.057, −0.008]	−0.037^**^	0.014	[−0.060, −0.014]
Others	−0.039^*^	0.016	[−0.064, −0.013]	−0.095^**^	0.035	[−0.15, −0.038]
W1 Parental Education (Ref: Medium)						
High	−0.099^**^	0.028	[−0.15, −0.052]	−0.10^**^	0.030	[−0.15, −0.053]
Low	0.84^***^	0.059	[0.74, 0.93]	0.83^***^	0.058	[0.73, 0.92]
W1 Annual Household Income Per Capita (Log)	−0.058^**^	0.019	[−0.088, −0.027]	−0.058^**^	0.019	[−0.088, −0.027]
W1 Parental Psychological Distress	0.035^*^	0.016	[0.009, 0.061]	0.024	0.015	[−0.001, 0.049]
W1 Single Parenthood	0.032^†^	0.017	[0.004, 0.059]	0.032^†^	0.016	[0.005, 0.059]
W1 Living with Grandparent(s)	0.044^**^	0.016	[0.018, 0.070]	0.047^**^	0.016	[0.021, 0.072]
W1 Having a Live-In Domestic Helper(s)	−0.028^†^	0.014	[−0.051, −0.004]	−0.027^†^	0.014	[−0.050, −0.004]
W1 Neighborhood Quality (Z-Score)	−0.022^†^	0.013	[−0.044, <0.001]	−0.020	0.014	[−0.043, 0.002]

*** *p* < 0.001;

** *p* < 0.01;

* *p* < 0.01;

† *p* < 0.10.

**Table 3:** Indirect effects of pre-pandemic parental resources in wave 1 (W1) on child internalizing problems during the COVID-19 pandemic in wave 2 (W2) and changes in child internalizing problems from W1 to W2.

Mediating Pathways	*β*	*SE_β_*	95% CI
Pathways to Child Internalizing Problems in W2 (Model 3)			
Total indirect effect of Verbal Cognitive Ability	−0.005	0.003	[−0.011, <0.001]
Parental Verbal Cognitive Ability → Positive Parental Control → (Fewer) Child Internalizing Problems	−0.007^*^	0.003	[−0.013, −0.003]
Total indirect effect of Self-Control	−0.016^**^	0.004	[−0.023, −0.008]
Parental Self-Control → (Less) Negative Parental Control → (Fewer) Child Internalizing Problems	−0.014^**^	0.004	[−0.018, −0.005]
Total indirect effect of Economic Stress	0.027^***^	0.007	[0.015, 0.039]
Economic Stress → (Less) Positive Parental Control → Child Internalizing Problems	0.009^*^	0.004	[0.004, 0.016]
Economic Stress → Negative Parental Control → Child Internalizing Problems	0.018^**^	0.006	[0.007, 0.024]
Pathways to Changes in Child Internalizing Problems from W1 to W2 (Model 4)			
Total indirect effect of Verbal Cognitive Ability	−0.009^*^	0.004	[−0.014, −0.003]
Parental Verbal Cognitive Ability → Positive Parental Control → Changes in Child Internalizing Problems	−0.008^*^	0.003	[−0.013, −0.003]
Total indirect effect of Self-Control	−0.016^**^	0.005	[−0.023, −0.008]
Parental Self-Control → (Less) Negative Parental Control → Changes in Child Internalizing Problems	−0.012^**^	0.004	[−0.019, −0.006]
Total indirect effect of Economic Stress	0.026^***^	0.007	[0.015, 0.037]
Economic Stress → (Less) Positive Parental Control → Changes in Child Internalizing Problems	0.010^*^	0.004	[0.003, 0.016]
Economic Stress → Negative Control → Changes in Child Internalizing Problems	0.015^**^	0.005	[0.007, 0.024]
Economic Stress → (Less) Positive Parental Control → (Lower) DoG → Changes in Child Internalizing Problems	0.001^†^	<0.001	[<0.001, 0.001]

Covariates included child age, gender, ethnicity, parental education, annual household income per capita, parental psychological distress, single parenthood, living with grandparents, having a live-in domestic helper(s), neighborhood quality, and pre-pandemic child internalizing problems.

*** *p* < 0.001;

** *p* < 0.01;

* *p* < 0.01; †*p* < 0.10.

## 4. Discussion

To our best knowledge, this was the first longitudinal study that used a large, nationally representative, and socioeconomically and ethnically diverse sample of young children in Asia to investigate the complex mechanism regarding how diverse environmental and child-level resources influence children’s internalizing symptoms. Based on the data gathered in Singapore, the current study examined (a) the longitudinal relations of multi-level resources to children’s internalizing problems and (b) the mediating pathways from parental resources to child internalizing problems through parental control and child self-regulation. The present work has addressed these questions in the context of COVID-19, and the findings can provide insight into the influences of social-ecological systems on Asian children’s emotional resilience under adverse and uncertain circumstances.

We discovered that primary caregivers’ verbal cognitive ability, self-control, and low economic stress were critical parental resources that predicted fewer child internalizing problems and greater emotional resilience directly or indirectly through parental control and child self-regulation. In particular, primary caregivers’ cognitive, psychological, and economic resources were related to more positive parental control (e.g., limit setting) and less negative parental control (e.g., harsh discipline), which predicted children’s fewer internalizing problems during the pandemic, and alleviated their increases in internalizing problems over time. Positive parental control also predicted children’s greater self-regulation during early childhood, which further diminished an exacerbation of internalizing problems. In addition, we examined other pre-pandemic family and community characteristics as covariates in this study. We discovered that parental psychological well-being (e.g., low psychological distress), family socioeconomic status (e.g., parental education and family income), living with two parents, living without grandparents, having a live-in domestic helper, and neighborhood quality also predicted children’s fewer internalizing problems during the pandemic. In particular, family socioeconomic status and living arrangements further predicted children’s changes in internalizing problems over time.

The first unique feature of this study is the systematic investigation of the longitudinal effects of multi-level resources on children’s outcomes in the same model. First, our research has filled the gaps in understanding the influences of parental resources in various forms on early childhood development. While previous studies largely focused on parental psychological distress and economic stress, we have taken into account parental cognitive and self-regulatory abilities. We discovered that cognitive verbal ability, self-control, and low economic stress could provide parents with cognitive, psychological, and economic resources to nurture children’s emotional well-being and resilience. Second, while positive parental control was less examined in prior research compared to punitive approaches and parental warmth, we have included different types of parental control in the same model. Our findings demonstrated that positive and negative parental control could exert considerably different effects on children’s emotional development. While negative parental control (e.g., harsh discipline) intensifies children’s internalizing problems, positive parental control (e.g., limit setting) can reduce children’s internalizing problems and enhance their emotional resilience. Third, we highlight the interplay between environmental and child-level factors. Our data shows that early childhood self-regulation serves as a crucial child-level resource that can be shaped by early environments and then empowers children to counteract the impacts of adversities on their mental health. Finally, we have considered the influences of other pre-pandemic family and community characteristics on early childhood development. As expected, primary caregivers’ psychological distress can have a long-term impact on children’s emotional symptoms; family socioeconomic status (including parental education and family income) provides the family with resources to foster children’s emotional well-being and resilience under adversity; children growing up in single-parent families have a higher risk of mental health issues and may experience a more significant increase in internalizing symptoms over time. Living arrangements can also play a part in early emotional development. For example, we found that having a live-in domestic helper can predict fewer child internalizing problems and less aggravation of internalizing problems over time, possibly due to the alleviated daily hassles in the family. Contrary to our hypothesis, living with grandparents can be longitudinally related to more child internalizing problems during the pandemic and a large increase in internalizing problems over time, possibly due to disagreements in parenting or intergenerational conflicts. At the child level, cultural backgrounds were also found to be associated with children’s emotional symptoms during the pandemic. Indian children had fewer internalizing problems than their Chinese and Malay counterparts, based on parent reports. This result may be explained by Indian families’ socialization goals and cultural values that view childhood as a carefree period (Rao et al., 2003). At the community level, neighborhood quality (e.g., safety and cohesion) can predict children’s fewer internalizing problems during the pandemic. Indeed, resilience outcomes are often observed in communities with higher social cohesion (Heid et al., 2016). Together, our findings highlight the roles of community-, family-, parent-, and child-level resources in shaping children’s positive development.

More importantly, the present research revealed the mechanism through which multi-level resources work together to promote children’s emotional well-being and resilience. Guided by the family stress theory and the model of self-regulation development, we established the mediating pathways linking three types of parental resources (e.g., cognitive, psychological, and economic resources) to children’s internalizing problems through two types of parental control (e.g., positive and negative control) and child self-regulation. The pathways remained significant after controlling for other family and community characteristics.

Among the three forms of parental resources, self-control and verbal cognitive ability were positively correlated, and they were both associated with lower economic stress. Indeed, self-control has been related to cognitive functions (McClelland et al., 2007; Shamosh and Gray, 2008) and verbal ability (Cole et al., 2003; Roben et al., 2013) since early childhood. Cognitive function and attention may support individuals’ ability to regulate their cognition, emotions, and behaviors consciously. Self-control also supports individuals to perform better in cognitive tasks. Moreover, self-control and verbal cognitive ability provide parents with knowledge and skills to manage financial matters in the family, communicate needs and thoughts, and plan for their expenditures in a future-oriented way. Thus, parents with greater self-control and verbal cognitive ability are less likely to experience economic stress. Parents who experience a lower level of stress may also practice self-control more frequently in their daily lives (e.g., inhibiting temptation, breaking bad habits, and regulating emotions) and perform better in cognitive tasks.

Furthermore, primary caregivers’ cognitive, psychological, and economic resources influence their parenting strategies. Our findings suggest that primary caregivers with stronger self-control and less economic stress are less likely to engage in negative parental control (e.g., harsh discipline or punishment). Primary caregivers with more advanced verbal cognitive ability and less economic stress tend to deploy positive parental control, such as setting limits on their children’s activities accompanied by discussions, guidance, and encouragement. The relationship between economic stress and negative parental control was in line with the family stress model, which posits that the experience of stress leads to more punitive and less nurturing parenting (Yeung et al., 2002; Conger and Donnellan, 2007). The relationship between economic stress and positive parental control (e.g., limit setting) was less well documented in the literature compared to harsh discipline and parental warmth. Our finding adds to the literature by revealing the differential roles of negative and positive parental control in the association between economic stress and child outcomes. Also, the relations of parents’ cognitive and self-regulatory abilities to parenting strategies were less explored in prior research. Our findings further advance the literature by illustrating that verbal cognitive ability and self-control can provide parents with knowledge and skills to employ more functional strategies to facilitate their children to regulate behaviors and emotions. Self-control enables parents to regulate their emotions effectively and inhibit the tendency to use emotionally charged strategies, such as spanking, grounding, and scolding. Verbal cognitive ability enables parents to use more advanced reasoning and rich vocabulary to guide their children to follow the rules and internalize the rules.

Parental resources and parenting behaviors create the most proximal environment for children’s early development of self-regulation. Corresponding to the model of self-regulation development (Kopp, 1982), our findings indicate that young children’s self-regulation can be nurtured by primary caregivers’ self-control, verbal cognitive ability, and positive parental control. Parents with strong self-control to resist temptation, inhibit unfavorable behaviors, and perform socially desirable behaviors can act as good role models when their children learn and practice regulating behaviors and emotions. Previous studies showed that parental expressivity, including nonverbal and verbal expressions of emotions, predicted children’s physiological and behavioral regulation (Liew et al., 2011). We argued that primary caregivers with better verbal cognitive skills could use more effective expressivity, instructions, and encouragement to guide their children to regulate their emotions and behaviors internally. Furthermore, positive parental control (derived from parents’ verbal cognitive ability and low economic stress) can further facilitate young children’s development of self-regulation. The relationship between positive parental control and child self-regulation aligned with previous meta-analysis results (Karreman et al., 2006). Using directiveness with low to moderate power assertion (e.g., setting rules, discussing the rules with children, and enforcing the rules) can facilitate children to internalize caregivers’ external control and progress to internal control (Calkins et al., 1998; Belsky et al., 2000; Kochanska et al., 2000, 2001). In contrast, excessively controlling children’s behaviors through harsh discipline without granting them sufficient age-appropriate autonomy results in children’s negative emotions (e.g., helplessness and lack of control) and poorer emotional regulation skills, which further lead to their internalizing symptoms, such as anxiety and depression (Hudson and Rapee, 2002; Bayer et al., 2006).

Finally, self-regulation serves as an essential child-level resource that empowers children to adjust to adversities and maintain or improve their mental health under adversity (Lengua and Long, 2002; Lengua, 2003; Eiden et al., 2007; Lengua et al., 2008; Flouri et al., 2014). Our study shows that when children have greater self-regulation (e.g., delayed gratification), they are less likely to intensify internalizing problems under stressful or challenging situations. The ability to delay gratification is an important aspect of self-regulation, reflecting one’s capacity to inhibit dominant responses and perform subdominant responses. Nurturing self-regulation during the early years can provide children with a good foundation to regulate their emotions and behaviors and to counteract the negative impacts of significant life events on their emotional well-being.

The current research has several theoretical implications. First, our findings fill the gaps in understanding the complex mechanisms regarding how various parental resources can protect children from internalizing symptoms through functional parental control and child self-regulation. The present work has incorporated the family stress model (Conger et al., 1994; Conger and Donnellan, 2007; Yeung et al., 2002) and the model of self-regulation development (Kopp, 1982) in a single comprehensive framework. More importantly, we extended these well-established theories from the Western, Educated, Industrialized, Rich, and Democratic (WEIRD) context to Asian cultures and from the non-COVID-19 context to the COVID-19 context. Second, this study has advanced the literature by illustrating how children’s emotional well-being and resilience can be nurtured by multi-level resources in children’s social-ecological systems, including community-, family-, parent-, and child-level resources. These findings have added to the social-ecological framework of resilience, which emphasizes individuals’ interactions with the environment (Bonanno, 2004; Ungar et al., 2013). In addition, based on the data collected from a large, national probability, socioeconomically and ethnically diverse sample of children in a multicultural Asian country, our findings have excellent generalizability. They can also shed light on social-emotional development and resilience among children from other countries with similar characteristics.

The present study also has significant practical implications. Our research calls for attention to nurturing children’s emotional resilience under stressful and uncertain circumstances by activating multi-level resources in their social-ecological systems. Consistent with previous studies on the interaction between child-level competence and environmental resources in the Asian context (e.g., Chen and Yang, 2022), our study underscores the interplay between external and internal resources. Resilience-based intervention programs have been effective in reducing internalizing symptoms and promoting psychological well-being among children and adolescents (see Dray et al., 2017 for a systematic review). It is necessary to design and implement appropriate interventions during the post-pandemic period to boost individual-level and environmental protective factors to foster children’s resilience. Moreover, our findings affirm the critical roles of family processes in early childhood development in different domains, including self-regulation, emotional well-being, and resilience. We recommend family-based interventions to enhance parents’ cognitive, psychological, and socioeconomic strengths and improve functional parent–child interactions so as to promote family resilience. These practices can further facilitate children to build a good foundation for positive adjustments to potential adversities or major life events. Last but not least, this study informs policymaking and highlights the importance of building human capital (such as health, knowledge, and skills) and community resources (such as neighborhood safety, social support, and cohesion) to empower families and individuals to improve their well-being and resilience during future times of adversity.

The interpretation of current findings must take into account the limitations of this study. First, most of the variables were collected by onsite self-report or informant-report measures, which may compromise the accuracy of the data due to social desirability (Mortel and Thea, 2008). In this study, only parental verbal cognitive ability and child self-regulation were assessed by behavioral measures, while other information was reported by primary caregivers. Future studies will benefit from deploying observations, behavioral measurements, and multi-informant reports to assess parents’ self-control, parent–child interactions, and children’s emotional symptoms. Relatedly, our findings primarily relied on maternal reports because 95% of the primary caregivers were mothers. The information about father–child interactions and father-reported family processes was minimal. Future studies may investigate the roles of fathers’ parenting in children’s social-emotional development in order to get a more comprehensive understanding of children’s emotional resilience from the social-ecological perspective. Lastly, the protective factors in this study were identified during early childhood, which is a critical stage for child development in many domains. The literature has documented some unique protective and risk factors (e.g., parent–child conflicts, peer relationships, social connections, media exposure, and concerns for governments’ restrictions) for adolescents’ mental health during the COVID-19 pandemic (Magson et al., 2021). Thus, when children enter adolescence, other resources in the social-ecological system should be incorporated to foster their emotional well-being and resilience.

## 5. Conclusion

The present research has identified an array of pre-pandemic protective factors against children’s internalizing problems during the COVID-19 pandemic. Parental resources (e.g., cognitive, psychological, and socioeconomic resources), functional parent–child interactions (e.g., more positive control and less negative control), child-level resources (e.g., self-regulation), family characteristics (e.g., living arrangements), and community characteristics (e.g., safety and cohesion) contribute to children’s mental health. Moreover, this study has helped illustrate the complex mechanisms regarding how parental resources can protect young children from declines in mental health under adversity through parental control and child self-regulation. It is critical to develop and implement resilience-based and family-based interventions to activate multi-level resources in young children’s social-ecological systems, so as to promote their resilience and psychological adjustment to future stressful or challenging circumstances.

## Data availability statement

The raw data supporting the conclusions of this article will be made available by the authors, without undue reservation.

## Ethics statement

The studies involving human participants were reviewed and approved by the Institutional Review Board (IRB) at the National University of Singapore. Written informed consent to participate in this study was provided by the participants’ legal guardian/next of kin.

## Author contributions

LC and W-JY contributed to the conception and design of the study. W-JY acquired the funding and resources for the study and supervised the project. LC organized the dataset, performed the statistical analysis, and wrote the first draft of the manuscript. All authors contributed to the article and approved the submitted version.

## Funding

This work was supported by the Ministry of Education, Singapore, under the Social Science Research Thematic Grant (MOE2016–SSRTG–044).

## Conflict of interest

The authors declare that the research was conducted in the absence of any commercial or financial relationships that could be construed as a potential conflict of interest.

## Publisher’s note

All claims expressed in this article are solely those of the authors and do not necessarily represent those of their affiliated organizations, or those of the publisher, the editors and the reviewers. Any product that may be evaluated in this article, or claim that may be made by its manufacturer, is not guaranteed or endorsed by the publisher.
