# Supplementary material for: Pre-pandemic family resources and child self-regulation in children’s internalizing problems during COVID-19: a multi-level social-ecological framework for emotional resilience
Source: Front Psychol. 2023 Jul 26;14:1203524. doi: 10.3389/fpsyg.2023.1203524 (PMC10410081; doi:10.3389/fpsyg.2023.1203524)
Supplement: Supplementary file 1 [file Table_1.DOCX]

Supplementary Material

Pre-Pandemic Family Resources and Child Self-Regulation in Children’s Internalizing Problems during the COVID-19 Pandemic: Understanding Singaporean Children’s Emotional Resilience

Luxi Chen*, Wei-Jun Jean Yeung

*** Correspondence:** Luxi Chen: luxichen@nus.edu.sg

# Appendix A. Parental Verbal Ability Test Items

Now I will show you some passages. Please read the test passages silently to yourself, and tell me what word belongs in the blank space.

1. Polar bears have heavy fur coats. They live where it is _______.
2. What is a flood? It’s a condition that exists when a river overflows its banks and the _______ spreads out elsewhere.
3. For generations, children have built many types of model airplanes. Although they are fun to make, few of them can actually ______.
4. Have you ever had to prepare a _____? Keep in mind that a good speaker will get the audience’s attention right from the start.
5. You’ll be amazed at how easy it is to master the techniques of moneymaking. You’ll see how to avoid the most dangerous mistakes people make with their _____.
6. Places hardest hit by winter storms are turning to sophisticated technology to clear roads of snow and ice. This innovative technology is making it much safer for ____.
7. The meeting last week was very _____. No one who attended would admit it had taken place, much less where.
8. In our modern world, the lifespan of many products is very short. What seems indispensable today becomes trivial tomorrow. Determining lasting _____ is very difficult.

# Appendix B. Behavior Problems Index (BPI) in Both Waves

**Supplementary Table 1. Factor Analysis (Principal Component Analysis with Varimax Rotation) Results of BPI in Wave 1** (*n* = 4,543)

| For the next set of statements, decide whether they are not true, sometimes true, or often true, of (CHILD)’s behavior. He/She… | Factor 1 | Factor 2 | Dimension |
| --- | --- | --- | --- |
| a. has sudden changes in mood or feeling. | **0.43** | 0.36 | **EXT** |
| d. cheats or tells lies. | **0.47** | 0.15 | **EXT** |
| f. argues too much. | **0.67** | 0.13 | **EXT** |
| g. has difficulty concentrating, cannot pay attention for long. | **0.44** | 0.40 | **EXT** |
| j. is disobedient. | **0.58** | 0.19 | **EXT** |
| k. does not seem to feel sorry after (he/she) misbehaves. | **0.45** | 0.29 | **EXT** |
| m. is impulsive, or acts without thinking. | **0.49** | 0.31 | **EXT** |
| q. is restless or overly active, cannot sit still. | **0.62** | 0.17 | **EXT** |
| r. is stubborn, sullen, or irritable. | **0.70** | 0.15 | **EXT** |
| s. has a very strong temper and loses it easily. | **0.68** | 0.15 | **EXT** |
| w. clings to adults. | **0.55** | 0.12 | **EXT** |
| x. cries too much. | **0.55** | 0.20 | **EXT** |
| y. demands a lot of attention. | **0.64** | 0.18 | **EXT** |
| b. feels or complains that no one loves him/her. | 0.25 | **0.44** | **INT** |
| c. is rather high strung, tense and nervous. | 0.30 | **0.53** | **INT** |
| e. is too fearful or anxious. | 0.31 | **0.50** | **INT** |
| h. is easily confused, seems to be in a fog. | 0.33 | **0.54** | **INT** |
| l. has trouble getting along with other children. | 0.19 | **0.62** | **INT** |
| n. feels worthless or inferior. | 0.13 | **0.65** | **INT** |
| o. is not liked by other children. | 0.16 | **0.64** | **INT** |
| p. has a lot of difficulty getting (his/her) mind off certain thoughts. | 0.34 | **0.53** | **INT** |
| t. is unhappy, sad or depressed. | 0.24 | **0.61** | **INT** |
| u. is withdrawn, does not get involved with others. | 0.14 | **0.68** | **INT** |
| aa. feels others are out to get (him/her). | 0.18 | **0.69** | **INT** |
| cc. is secretive, keeps things to (himself/herself). | 0.15 | **0.64** | **INT** |
| dd. worries too much. | 0.07 | **0.68** | **INT** |
| i. bullies or is cruel or mean to others. ^a^ | 0.36 | 0.34 | **EXCLUDED** |
| v. breaks things on purpose or deliberately destroys (his/her) own or another's things. ^b^ | 0.33 | **0.45** | **EXCLUDED** |
| z. is too dependent on others. ^a^ | 0.39 | 0.38 | **EXCLUDED** |
| bb. hangs around with kids who get into trouble. ^b^ | 0.15 | **0.58** | **EXCLUDED** |
| **Cronbach's *α*** | **0.86** | **0.88** | **0.92** |
| **N of items** | **13** | **13** | **26** |

*Note*. EXT=externalizing problems. INT=Internalizing problems. Value in bold indicated acceptable factor loading. ^a^The item is excluded due to low factor loading (< .40) in both dimensions. ^b^The item is excluded because its factor loading was inconsistent with its theoretical construct.

**Supplementary Table 2. Factor Analysis (Principal Component Analysis with Varimax Rotation)** **Results of BPI in Wave 2** (*n* = 2,953)

| For the next set of statements, decide whether they are not true, sometimes true, or often true, of (CHILD)’s behavior. He/She… | **Factor 1** | **Factor 2** | **Dimension** |
| --- | --- | --- | --- |
| a. has sudden changes in mood or feeling. | **.490** | .225 | **EXT** |
| f. argues too much. | **.584** | .138 | **EXT** |
| g. has difficulty concentrating, cannot pay attention for long. | **.472** | .215 | **EXT** |
| j. is disobedient. | **.626** | .081 | **EXT** |
| k. does not seem to feel sorry after (he/she) misbehaves. | **.506** | .163 | **EXT** |
| m. is impulsive, or acts without thinking. | **.541** | .296 | **EXT** |
| q. is restless or overly active, cannot sit still. | **.617** | .146 | **EXT** |
| r. is stubborn, sullen, or irritable. | **.668** | .199 | **EXT** |
| s. has a very strong temper and loses it easily. | **.668** | .133 | **EXT** |
| v. breaks things on purpose or deliberately destroys (his/her) own or another's things. | **.447** | .257 | **EXT** |
| w. clings to adults. | **.499** | .151 | **EXT** |
| x. cries too much. | **.512** | .148 | **EXT** |
| y. demands a lot of attention. | **.644** | .124 | **EXT** |
| z. is too dependent on others. | **.508** | .260 | **EXT** |
| b. feels or complains that no one loves him/her. | .273 | **.444** | **INT** |
| c. is rather high strung, tense and nervous. | .256 | **.565** | **INT** |
| e. is too fearful or anxious. | .221 | **.566** | **INT** |
| h. is easily confused, seems to be in a fog. | .298 | **.477** | **INT** |
| l. has trouble getting along with other children. | .164 | **.511** | **INT** |
| n. feels worthless or inferior. | .123 | **.619** | **INT** |
| o. is not liked by other children. | .137 | **.561** | **INT** |
| p. has a lot of difficulty getting (his/her) mind off certain thoughts. | .323 | **.508** | **INT** |
| t. is unhappy, sad or depressed. | .317 | **.508** | **INT** |
| u. is withdrawn, does not get involved with others. | .152 | **.596** | **INT** |
| aa. feels others are out to get (him/her). | .279 | **.528** | **INT** |
| cc. is secretive, keeps things to (himself/herself). | .119 | **.426** | **INT** |
| dd. worries too much. | .103 | **.539** | **INT** |
| d. cheats or tells lies. ^a^ | .379 | .199 | **EXCLUDED** |
| i. bullies or is cruel or mean to others. ^a^ | .371 | .256 | **EXCLUDED** |
| bb. hangs around with kids who get into trouble. ^b^ | .043 | **.601** | **EXCLUDED** |
| **Cronbach's *α*** | **0.85** | **0.83** | **0.90** |
| **N of items** | **14** | **13** | **27** |

*Note*. EXT=externalizing problems. INT=Internalizing problems. Value in bold indicated acceptable factor loading. ^a^The item is excluded due to low factor loading (< .40) in both dimensions. ^b^The item is excluded because its factor loading was inconsistent with its theoretical construct.
